# Experience of management of pediatric upper gastrointestinal perforations: a series of 30 cases

**DOI:** 10.3389/fped.2023.1261336

**Published:** 2023-10-11

**Authors:** Mengqi Wang, Shuai Sun, Qiong Niu, Baoguang Hu, Haiyan Zhao, Lei Geng, Tingliang Fu, Hong Qin, Bufeng Zheng, Hesheng Li

**Affiliations:** ^1^ Department of Pediatric Surgery, Binzhou Medical University Hospital, Binzhou, China; ^2^ Department of Gastroenterology, Binzhou Medical University Hospital, Binzhou, China; ^3^ Department of Gastrointestinal Surgery, Binzhou Medical University Hospital, Binzhou, China; ^4^ Department of Colorectal Surgery, Binzhou Medical University Hospital, Binzhou, China; ^5^ Department of General Surgery, Children’s Hospital Affiliated to Shandong University, Jinan, China

**Keywords:** upper gastrointestinal perforation, peptic ulcer, diagnosis, surgical management, children

## Abstract

**Background:**

This study aimed to explore the characteristics of pediatric upper gastrointestinal (UGI) perforations, focusing on their diagnosis and management.

**Methods:**

Between January 2013 and December 2021, 30 children with confirmed UGI perforations were enrolled, and their clinical data were analyzed. Two groups were compared according to management options, including open surgical repair (OSR) and laparoscopic/gastroscopic repair (LR).

**Results:**

A total of 30 patients with a median age of 36.0 months (1 day–17 years) were included in the study. There were 19 and 11 patients in the LR and OSR groups, respectively. In the LR group, two patients were treated via exploratory laparoscopy and OSR, and the other patients were managed via gastroscopic repair. Ten and three patients presented the duration from symptom onset to diagnosis within 24 h (*p* = 0.177) and the number of patients with hemodynamically unstable perforations was 4 and 3 in the LR and OSR groups, respectively. Simple suture or clip closure was performed in 27 patients, and laparoscopically pedicled omental patch repair was performed in two patients. There was no significant difference in operative time and length of hospital stay between the LR and OSR groups. Treatment failed in two patients because of severe sepsis and multiple organ dysfunction syndrome, including one with fungal peritonitis.

**Conclusion:**

Surgery for pediatric UGI perforations should be selected according to the general status of the patient, age of the patient, duration from symptom onset, inflammation, and perforation site and size. Antibiotic administration and surgical closure remain the main strategies for pediatric UGI perforations.

## Introduction

Upper gastrointestinal (UGI) perforation is a life-threatening condition. Although UGI perforation is uncommon in the pediatric population (1), its etiology is multifaceted and includes inflammation (2), congenital defects of gastric musculature or spontaneous rupture (3, 4), trauma (5), peptic or drug-induced ulcer (5, 6), ingestion of sharp foreign bodies or high-powered magnets (e.g., buckyballs) (7, 8), and iatrogenic injury (9–11).

Due to effective acid suppression, peptic gastroduodenal ulcer perforation has become less prevalent (11, 12), and endoscopic management of surgical gastrointestinal diseases has rapidly increased with advancements in endoscopic techniques over the last decade. Thus, the risk of iatrogenic perforation related to endoscopic procedures for UGI may increase (9–11).

The diagnosis of UGI perforation is based on medical history, physical examination, laboratory investigation, imaging studies, and endoscopy (13, 14). However, managing pediatric UGI perforation, including non-surgical and surgical treatments, remains a challenge in clinical practice. The general principles for these severe conditions are prompt diagnosis and effective treatment, although there is little standardization due to varied etiologies, different perforation sites and sizes, various clinical scenarios, and varied diagnostic methods (15, 16).

Herein, we retrospectively review the clinical data of UGI perforations in pediatric patients and focus on their etiologies, diagnostic methods, and management options. This study aimed to provide additional information on the diagnosis and management of pediatric UGI perforations.

## Patients and methods

The present study included 30 pediatric patients with surgically confirmed UGI perforations at the Binzhou Medical University Hospital and Children's Hospital Affiliated to Shandong University from January 2013 to December 2021. Patients with UGI perforations of various etiologies were included in the study. The inclusion criteria are as follows: patients aged ≤18 years with gastroduodenal perforations confirmed by surgery or endoscopy. The exclusion criteria are as follows: patients in whom UGI perforations were clinically suspected but unconfirmed via surgery or endoscopy and aged >18 years.

The patients were then divided into two groups according to the different surgical approaches: laparoscopic repair and traditional open repair groups. Clinical data, such as age, sex, medical history, clinical signs, diagnostic modalities, surgical approach, intraoperative findings, operative time, postoperative complications, and length of hospital stay, were collected and compared between the groups. Postoperative complications were divided into grades I–IV according to the Clavien–Dindo classification (17).

The study adhered to the ethical principles of the Declaration of Helsinki and the local ethical and legal requirements. Written informed consent was provided by the legal guardians of the patients. According to the Institutional Review Board of Binzhou Medical University, formal approval was not required for retrospective archived studies.

Statistical analyses were conducted using descriptive statistics, Student's *t*-test, and the chi-squared test, where appropriate. Statistical significance was set at *p* < 0.05. Statistical analyses were performed using SPSS (version 27.0; SPSS Inc., Chicago, IL, USA).

## Results

As shown in Table 1, 30 patients with gastroduodenal perforations were included, of whom 21 were males (70.0%) and nine were females (30.0%), with a median age of 3.0 (0–17) years. Seventeen patients (56.7%) were 3 years of age. The etiologies included peptic ulcers (*n* = 9), foreign body ingestion (*n* = 11, including five patients with multiple buckyballs), congenital malformation (*n* = 2), trauma (*n* = 2), iatrogenic cause (possible electrosurgical knife induced injury, *n* = 1), necrotizing enterocolitis (NEC, *n* = 1), and unknown causes (*n* = 4). Of the nine patients with perforated peptic ulcer (PPU), the *Helicobacter pylori* test was conducted in four patients and was positive in two (both males, aged 12 and 15 years). Eighteen patients underwent laparoscopic repair, 11 underwent open repair, and one underwent gastroscopic closure. Eight (42.1%) and nine (81.2%) patients in the laparoscopic/gastroscopic repair (LR) and open surgical repair (OSR) groups (*p* = 0.034), respectively, were ≤3 years of age, indicating a tendency to undergo open repair in children aged ≤3 years.

**Table 1:** Characteristics, diagnosis, management, and outcome of pediatric upper gastrointestinal perforation.

Groups	Laparoscopic/gastroscopic repair (*n*)	%	Open repair (*n*)	%	*p*-Value
Number of patients	19^a^		11		
Age (medium, months)					0.034
≤36	8	42.1	9	81.8	
>36	11	57.9	2	18.2	
Gender					
Male	15	78.9	6	54.5	
Female	4	21.1	5	45.5	
Time to admission [median (min. max.) hours]	1–120		12–120		0.177
≤24 h	10	52.6	3	27.3	
>24 h	9	47.4	8	72.7	
Shock	4	21.1	3	27.3	0.151
Etiology					
Peptic ulcer^c^	9^b^	47.4	0		
Foreign body ingestion	7^b^	36.8	4	36.4	
Congenital	1	5.3	1	9.1	
Iatrogenic	0		1	9.1	
Trauma	1	5.3	1	9.1	
Concomitant with NEC	0		1	9.1	
Idiopathic	1	5.3	3	27.3	
Lab investigations					
CRP (mg/L)	9.42 ± 39.42		41.14 ± 40.64		0.183
Serum natrium (mmol/L)	137.24 ± 7.76		136.73 ± 15.27		0.831
Serum albumin (g/L)	40.24 ± 12.63		42.79 ± 59.91		0.722
Imaging (free air)					
Plain x-ray	10		5		
Ultrasonography	6		8		
CT scan	7		0		
Perforation site					
Fundus of the stomach	2	10.5	1	9.1	
Body of the stomach	8	42.1	6	54.6	
Gastric antrum	3	15.8	0	0	
Pylorus + gastric fundus	0		1	9.1	
Duodenum	6	31.6	3	27.3	
Perforation size (mm)	7.56 ± 17.44		9.67 ± 20.33		0.503
Admission to surgery [hours- median (min. max.)]	2 h–168 h		2 h–27 h		0.556
≤6	13	68.4	9	81.8	
>6	6	31.6	2	18.2	
Surgical procedure					
Simple suture	16	84.2	11	100	
Omental patch repair	2	10.5	0	0	
Gastroscopic clip closure	1	5.3			
Operative time (min)	212.62 ± 162.62		145.73 ± 69.00		0.114
Postop. complications^d^					
I	1		0		
II	0		1		
IIIb	1		0		
Hospital stay (days)	13.80 ± 15.20		16.08 ± 18.92		0.386

^a^ Two cases were converted to an open procedure.

^b^ One case was converted to an open procedure.

^c^ *Helicobacter pylori*-positive (urea breath testing for active infection).

^d^ Clavien–Dindo classification (17).

Almost all the patients presented with abdominal pain, tenderness, and rebound tenderness on palpation. Four and three patients experienced hypotension on admission in the LR and OSR groups (*p* = 0.151), respectively; the detailed data are shown in Table 2. The mean C-reactive protein level on admission was 9.42 ± 39.42 mg/L and 41.14 ± 40.64 mg/L in the LR and OSR groups (*p* = 0.183), respectively. The mean values of serum albumin and natrium levels were within the normal range between the two groups, although some patients had hypoalbuminemia and hyponatremia.

**Table 2:** Characteristics of cases with shock on admission.

Variables	*n* = 7
Age (year)	
≤3	4
>3	3
Gender	
Male	3
Female	4
Onset to admission (h)	
≤48	4
>48	3
Perforation site	
Gastric fundus	4
Gastric body	1
Gastric fundus + pylorus	1
Duodenum	1
Bacterial culture	
Peritoneal fluid	1
*Spores of Bacillus subtilis*	1
*Yeast; Escherichia coli*	2
*Candida albicans*	2
Negative	1
Sputum	
*Klebsiella pneumoniae*	
Antibiotics	
Meropenem combined with vancomycin	3
Linezolid	2
Tienam	1
Sulperazon combined with metronidazole	1
Mean length of hospital stay (day)	20.6
Outcome (survival rate)	5 (71.4%)

Plain radiography, ultrasonography, and CT scanning, or plain radiography plus ultrasonography/CT scan, were chosen for the two groups. The pneumoperitoneum sign helped make this diagnosis. In the LR group, the perforation sites were located on the fundus of the stomach (*n* = 2), body of the stomach (*n* = 8), gastric antrum (*n* = 3), and duodenum (*n* = 6); in the OSR group, the perforation sites were located on the fundus of the stomach (*n* = 1), body of the stomach (*n* = 7), and duodenum (*n* = 3). The two groups had no significant difference in the perforation sizes (7.56 ± 17.44 mm vs. 9.67 ± 20.33 mm, respectively, *p* = 0.503).

Of the seven patients with shock, three were males and four were females. Their ages ranged from 2 days to 11 years, with a median age of 2 years. The time until treatment initiation was <48 h (*n* = 4) or >48 h (*n* = 3). The perforation sites included the gastric fundus (*n* = 4), gastric body (*n* = 1), pylorus and fundus (*n* = 1), and duodenum (*n* = 1). Bacterial culture of the peritoneal fluid showed growth of *Candida albicans* (*n* = 2), spores of *Bacillus subtilis* yeast (*n* = 1), *Escherichia coli* (*n* = 1), and no growth (*n* = 2). Sputum cultures showed *Klebsiella pneumoniae* growth (*n* = 1). The patients were initially treated with antibiotics, including meropenem combined with vancomycin (*n* = 5), linezolid (*n* = 2), tienam (*n* = 1), and sulperazon combined with metronidazole (*n* = 2). The mean length of the hospital stay was 20.6 days. The survival rate of the patients with septic shock upon admission was 71.4%.

After initial fluid resuscitation, oxygen therapy, and intravenous antibiotics, surgical treatments were performed within 6 h of admission in 13 of 19 patients (68.4%) and nine of 11 (81.8%) patients in the LR and OSR groups (*p* = 0.556), respectively. In the LR group, perforation closure was performed via a simple suture (*n* = 16), omental patch repair (*n* = 2), and gastroscopic closure using clips (*n* = 1). In the OSR group, all 11 patients underwent simple suturing. The operative time was 212.62 ± 162.62 and 145.73 ± 69.00 min in the LR and OSR groups (*p* = 0.114), respectively.

Of the 28 patients who recovered, three patients experienced postoperative complications. Grades I (*n* = 1) and IIIb (*n* = 1) were observed in the LR group, and grade II (*n* = 1) was observed in the open repair group. The length of hospital stay was 13.80 ± 15.20 and 16.08 ± 18.92 days in the LR and OSR groups (*p* = 0.386), respectively. Treatment failed in two patients due to severe sepsis and multiple organ dysfunction syndrome (MODS), including one patient with intraperitoneal *C. albicans* infection.

## Discussion

Pediatric UGI perforation is an uncommon but severe disorder that needs to be diagnosed and treated immediately to improve the outcomes of the patients (15). Many causes, such as high-power magnets, congenital malformations, trauma, peptic ulcers, *H. pylori* infection, non-steroidal anti-inflammatory drugs, and iatrogenic injury, can lead to UGI perforation (2–5, 7–10, 13). The etiology of UGI perforation in our case series included almost all the causes mentioned above.

Pediatric PPU typically occurs in adolescents. However, in our case series, among the nine PPU patients, four (44.44%) were younger than 7 years, suggesting a tendency for a younger-age population. In the present study, UGI perforation predominantly occurred in boys (70%), which is similar to that reported in the literature (18). However, four of the seven patients with shock on admission were females (57.14%). This phenomenon requires further investigation of sex-based differences in clinical presentation and management strategies for pediatric UGI perforation (18, 19).

Regarding the option of imaging tools for diagnosing UGI perforation, pneumoperitoneum via plain abdominal radiography or CT scanning was observed in all patients, indicating a higher sensitivity of these two imaging modalities. Ultrasonography, a commonly used tool for differential diagnosis in pediatric acute abdomen, was performed in approximately half of the patients. The results showed a higher sensitivity of accurate diagnosis; this may be because highly skilled sonographers performed the ultrasonography (16). Ultrasonographic examination may reveal gas accumulation or free air in front of the liver, discontinuous gastroduodenal wall, swollen soft tissue, and free ascites (16, 20). If radiograms present no free gas, UGI perforation could not be ruled out. Point-of-care ultrasonography or double-contrast CT helps make a more accurate diagnosis (13, 16, 20, 21).

Regarding the management of UGI perforation, surgical intervention is relevant for the pediatric population, especially for those with intra-abdominal infections, sepsis, and unstable hemodynamics (1, 16, 22–24). In patients with unstable vital signs, emergent surgical intervention is performed after initial fluid resuscitation, oxygen therapy, and intravenous antibiotics. The postoperative survival rate was 71.4% in the patients with septic shock upon admission. This revealed that broad-spectrum antibiotics should be started immediately upon admission and continued during and after the surgical procedure (25–27). Antibiotics should be broad-spectrum, covering all possible pathogens. Multidrug regimens and appropriate dosing to ensure sufficient coverage and peak blood levels of antibiotics may play an important role in preventing the development of MODS (28–30). In the present study, the bacterial isolates were susceptible to vancomycin and linezolid. However, antibiotics can lead to fungal colonization and invasion across the mucosal barrier, causing fatal fungal peritonitis (31, 32). Empiric antifungal therapy is only considered in patients at a higher risk of fungal infection. Antifungals, such as echinocandins or liposomal amphotericin B, should also be considered in patients with a perforated abdominal viscus (29, 33). In this case series, fungal peritonitis was diagnosed in two patients, one of whom survived.

Conservative treatment is confirmed as safe and feasible for some selected cases of perforated peptic duodenal ulcers, including the duration of symptom onset being within 24 h of admission with a stable condition, localized peritoneal irritation signs, and mild ascites (15, 34, 35). A gastroduodenogram is usually needed to evaluate water-soluble contrast extravasation. If there is no contrast extravasation, it is suggestive of a self-sealed microperforation (12, 34). Endoscopic repair is another option for UGI perforation owing to its minimal invasiveness. However, upper endoscopic closure is generally performed within 24 h of duration from the symptom onset (20, 36–39). In the present study, only one patient (aged 2 years) with perforation of the stomach body due to misingestion of multiple magnet beans underwent endoscopic closure. We chose an endoscopic approach to seal the perforation because the lesion was induced by ingestion of multiple magnets, which is usually complicated by unmarked inflammation of the surrounding tissues. In a recent randomized controlled trial, Negm et al. (40) recommended that the indication for endoscopic repair of acute PPU is decontamination, that is, early chemical peritonitis and no septic shock. They concluded that endoscopic closure techniques, including clip technique accompanied by interventional radiological drainage, are effective for adult PPU. Bingener et al. (41) described a new natural orifice transluminal endoscopic surgery (NOTES) for closure of PPU in select cases with promising results, especially for the elderly and/or immunocompromised patients. Many challenges remain for endoscopic repair of GIT perforation; technical aspects and patient selection are still evolving (41). Endoscopic-guided GIT perforation repair may contribute to the future management of GIT perforation in the pediatric population, especially in small children. Prospective multicenter clinical trials are needed to prove the feasibility and efficiency of endoscopic closure in managing pediatric UGI perforation.

Surgical approaches include open or laparoscopic repair, which is based on the age of the patient (≤3 years), general condition, duration from symptom onset to diagnosis, hemodynamic status, comorbidities, perforation site and size, and experience and preference of the surgeon (21, 23, 36). In the LR group, the duration from symptom onset to operation in half of the patients was within 24 h, while in the OSR group, only 27.3% of the patients underwent operation within 24 h. Four patients with shock on admission underwent laparoscopic repair, and one patient had complications of postoperative gastric fistula and MODS. Unstable hemodynamics on admission may not be an absolute contraindication (42). Prospective studies are needed to provide available information on the safety and efficacy of laparoscopic repair in this group of patients (42–47).

The most common surgical procedure for UGI perforation is perforation closure via simple suturing (15, 19, 42). In the present case series, simple suturing was performed in most cases in both groups; only two patients underwent laparoscopically pedicled omental patch repair. The simple suture technique to repair the PPU is usually recommended because of its feasibility and low procedure time (12, 15, 42). Pedicled omental patch repair is another favorable technique, especially in cases with a friable edge or a large perforation, which cannot allow the approximation of perforation edges (20, 48–50). Regarding postoperative management for those PPU with *H. pylori* infection, proton pump inhibitors and eradication of *H. pylori* infection are essential to reduce peptic ulcer recurrence (40, 51, 52).

The limitations of the present study are as follows: the retrospective study design is flawed because of its unavoidable selection bias; the incidence of UGI perforation was low, and the sample size was small; prospective clinical trials using standardized diagnosis and management protocols are needed to improve the outcome of pediatric UGI perforations.

In conclusion, the etiology of pediatric UGI perforations is multifaceted. The diagnosis was mainly based on medical history, physical examination, laboratory and imaging investigations, including plain radiography, ultrasonography, and/or CT scanning. Treatment options vary according to the patient's general status, patient’s age, duration from symptom onset to diagnosis, pathological findings, and perforation sites and sizes. Surgeons are concerned about the safety of endoscopic perforation repair. Therefore, surgical assistance may be required in such cases. With increased experience in applying titanium clips and the nylon rope purse-suture technique, endoscopic repair with fewer complications can be accomplished in select cases (46, 53). Closure of UGI perforations via the simple suture technique combined with appropriate antimicrobials remains the mainstay of management in the pediatric population (54).

## Data Availability

The raw data supporting the conclusions of this article will be made available by the authors without undue reservation.
